# The Association Between Perceived Risk of COVID-19, Psychological Distress, and Internet Addiction in College Students: An Application of Stress Process Model

**DOI:** 10.3389/fpsyg.2022.898203

**Published:** 2022-06-20

**Authors:** Biru Chang, Jianhua Hou

**Affiliations:** ^1^School of Preschool Education, Xi’an University, Xi’an, China; ^2^Department of Social and Behavioural Sciences, City University of Hong Kong, Kowloon, Hong Kong SAR, China

**Keywords:** COVID-19, perceived risk, internet addiction, psychological distress, college students

## Abstract

The closed-off management of the university during coronavirus disease 2019 (COVID-19) may be associated with an elevated odds of psychological and behavioral issues among college students. We aimed to use the stress-process model to explore the potential mechanisms for this phenomenon. A total of 924 college students were recruited *via* posters, peer referrals, and class attendance. Among them, 82 (9%) were probable depression, 190 (20.8%) were probable anxiety, and 69 (7.5%) were internet addiction. Parallel mediation was used to test this theoretical model. For personal resources, the perceived risk of COVID-19 was positively associated with psychological distress *via* negative coping style (β = 0.051) and internet addiction *via* negative coping style or self-esteem (β = 0.023 for negative coping style, β = 0.015 for self-esteem). For social resources, the perceived risk of COVID-19 was positively associated with psychological distress and internet addiction *via* roommate relationships (β = 0.19 for psychological distress, β = 0.046 for internet addiction). Negative coping styles and roommate relationships are possible psychological mechanisms linking the perceived risk of COVID-19, psychological distress, and internet addiction.

## Introduction

In January 2022, the COVID-19 delta variant broke out in Xi’an city, Shaanxi province, China. Considering its high transmission and mortality rate, the local government immediately activated the new variant’s first-level public health emergency response. Xi’an went into partial lock-down from broke-out to January 23rd to curb population flow and contain the outbreak. Many college students were required to stay on campus immediately, disconnected from the outer world.

Emerging adulthood represents a critical development stage of neuro- and psychological maturity (Murray, 2018). However, the COVID-19 delta variant imposes unique challenges to this population. First, the delta-caused morbidity and mortality rate is much higher than other variants in the young population (Gurdasani et al., 2021). Second, considering its high transmission, delta-variant may severely disrupt their current study plan, job finding, and further study. A higher level of the perceived risk of COVID-19 is associated with a higher level of psychological distress and internet addiction in college students (Li et al., 2021; Wang et al., 2021). However, only a few studies have explored potential psychological mechanisms (Yıldırım et al., 2020).

The stress process model has provided a theoretical framework for sociological research into the effects of stress on psychological wellbeing (Pearlin et al., 1981; Aneshensel and Mitchell, 2014). The core components in this model are the stressors resources (personal and social resources, as mediators) and psychological distress (Pearlin et al., 1981). This model has been widely used and tested in a diverse population (e.g., Gayman et al., 2017; Choi et al., 2019). In this model, stress can be witnessed as arising from the occurrence of stressful events and the continuous problems (Pearlin et al., 1981). During COVID-19, although most students are free from COVID-19 infection, they may still perceive COVID-19 as a threat or be influenced *via* stress contagion (Yang et al., 2021). Thus, this perceived threat may be the core source of stress for colleague students at this specific time point. Resources, in this model, refer to the behaviors, perceptions, and cognitions used to alter the stressful situations or mediate the detrimental effect of stress, which are later divided into two categories: personal and social resources (Pearlin et al., 1981; Aneshensel and Mitchell, 2014). Psychological distress can be regarded as the emotional manifestation of stress, while internet addiction is the behavioral manifestation of stress. Previous studies have found that various sources of stress (e.g., academic stress or life stress, or COVID-19 threat) are associated with higher odds of internet addiction among college students (Jun and Choi, 2015; Li et al., 2016; Karakose, 2022). However, rare studies have considered and combined these concepts in the stress process model.

For personal resources, coping style represents a set of strategies that eliminate and avoid stress proliferation and alter the meaning of stressor (Pearlin and Bierman, 2013). Previous studies found that positive coping styles are associated with lower odds of psychological distress and internet addiction in both community and clinical samples during COVID-19 (Fu et al., 2020; Rogowska et al., 2020; Yu et al., 2020). Self-esteem, as an internal personal resource, is a set of attitudes toward one’s thoughts, feelings, meanings, and merits as a human being (Rosenberg, 1965; Swann and Seyle, 2006), which is crucial for a broad spectrum of internalizing problems (e.g., depression and anxiety) and externalizing problems (e.g., substance abuse and violence) (Mann et al., 2004). However, only a few studies have investigated the mediating role of self-esteem in linking stress and psychological distress during COVID-19 (Rossi et al., 2020; Lee et al., 2021). College students became vulnerable to losing self-esteem due to the enduring presence of noxious situations (e.g., prolonged closed-off management period) and their incapability to alter the situations (e.g., coerced closed-off management).

For social resources, social support refers to emotional, informational, and functional functions performed for a person by significant others (House et al., 1985). Social support is directly associated with mental health and buffers the detrimental effect of a stressor (Guilaran et al., 2018; Bender et al., 2019). The closed-off management and self-isolation may narrow college students’ availability or reception of social supports from parents and other social members. Thus, this public health strategy may flatten and contain the epidemic trend while simultaneously leading to negative psychological consequences. Another resource, especially important for quarantined college students, is roommate relationships because they are mandatorily quarantined in the dormitory with their roommates (Erb et al., 2014). Roommate relationships have demonstrated a wide range of buffering effects for depressive symptoms, anxiety symptoms, and substance abuse among college students (Guassi Moreira et al., 2016; Xu and Shrout, 2018).

**FIGURE 1:**
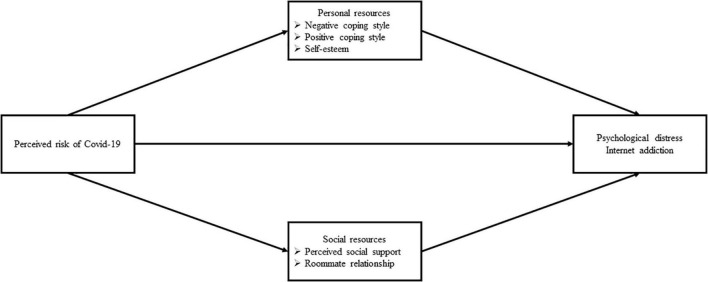
The conceptual diagram of the parellel mediation model.

This study aims to apply this stress process model to explore the association between perceived risk of COVID-19, psychological distress, and internet addiction among college students who are mandatorily staying on campus. We hypothesized that the perceived risk of COVID-19 was negatively associated with positive coping styles, global self-esteem, perceived social support, and roommate relationships, which, in turn, were negatively associated with psychological distress (H1–H4 for each mediation in order) and internet addiction (H6–H9 for each mediation in order). Moreover, the perceived risk of COVID-19 was positively associated with negative coping styles, which, in turn, were positively associated with psychological distress (H5) and internet addiction (H10). The conceptual diagram of the parallel model is shown in Figure 1.

## Materials and Methods

### Participants

All participants were recruited *via* posters, peer referral, and class attending and received a gift card (= 3 dollars) from January 3 to January 10, 2022. The analyzed sample is obtained using a convenient sampling method. To minimize potential bias, we put up research posters in each dormitory building, asked those who have participated in our survey to refer to other students, and asked at least one teacher of each department (11 departments regarding arts and humanity and 4 departments regarding science) to do surveys in their classes. The study ethics was reviewed and approved by the institutional review board of Xi’an University by expediting mode. We applied the list-wise deleting method to handle missing data considering that missing data is less than 10% for all models. Informed consent was obtained from all individual participants included in the study.

### Measures

#### Perceived Risk for COVID-19

The level of perceived risk for COVID-19 was measured by The Public Risk Perception Scale (Cronbach’s alpha = 0.885) (Yajun et al., 2020). This scale comprises 10 five-point items for four dimensions (the severity of the pandemic, the controllability, the severity of health impact, and the transmission possibility).

#### Coping Style

The coping style was measured by the Chinese version of the Simplified Coping Style Questionnaire (SCSQ) (Cronbach’s alpha = 0.737–0.811 in each subscale) (Xie, 1998). This scale comprises 20 items regarding different ways of coping with stressful events, including positive and negative coping styles with four choices (never used, occasionally used, sometimes used, and often used).

#### Global Self-Esteem

The level of global self-esteem was measured by a single-item self-esteem scale (SISE) (Robins et al., 2001), the reliability, and validity of which are similar to those of the 10-item Rosenberg Self-Esteem Scale.

#### Perceived Social Support

The level of perceived social support from teachers and family members was measured by the Perceived Social Support Scale (PSSS) (Zimet et al., 1988). This scale consists of 12 seven-point items (Cronbach’s alpha = 0.87).

#### Roommate Relationship

The level of roommate relationship was measured by the Roommate Relationship Diagnosis Scale (Cronbach’s alpha = 0.892) (Rong, 2005). This scale comprises 28 seven-point items for four dimensions (roommate communication, daily interaction, roommate support, and attitudes toward roommate and their behaviors).

#### Psychological Distress

The severity of anxiety symptoms was measured by the Chinese version of the Coronavirus Anxiety Scale (CAS) (Cronbach’s alpha = 0.87) (Chen et al., 2021). This scale comprises 5 five-point items ranging from 0 = not at all, to 4 = nearly every day. The cutoff score for probable anxiety was 9 for this scale in a Chinese community adult sample (Chen et al., 2021). The severity of depressive symptoms was measured by the Chinese version of the Patient Health Questionnaire (PHQ) (Cronbach’s alpha = 0.84) (Tsai et al., 2014). This scale comprises 9 four-point items ranging from 0 = not at all, to 4 = nearly every day. The cut-off score for probable depression was 10 on this scale in a Chinese community adult sample (Tsai et al., 2014). We calculated the level of psychological distress by summing the *Z*-score of two scales.

#### Internet Addiction

The severity of internet addiction was measured by the Revised Chen Internet Addiction Scale (Cronbach’s alpha = 0.92) (Mak et al., 2014). This scale comprises 26 four-point items for compulsive use, withdrawal, tolerance symptoms of Internet addiction, interpersonal and health-related problems, and time management problems. Respondents who scored ≥64 were classified as addictive internet users (Ko et al., 2005).

### Statistical Analyses

Firstly, we presented the mean, standardized deviation, and Pearson correlation for the predictor (perceived risk for COVID-19), mediators (positive coping style, negative coping style, self-esteem, perceived social support, and roommate relationship), and outcomes (psychological distress and internet addiction). Secondly, we adopted a parallel mediation to model the role of personal resources (positive, negative coping style, and self-esteem) and social or relational resources (social support from teachers or family members and roommate relationships). To establish the significance of the indirect effect, we examined the bootstrapped 95% confidence interval (CI) of the indirect effect with 5,000 repetitions. If the bias-corrected bootstrapped 95% CI does not contain zero, this supports the presence of the mediation effect (Hayes, 2017). All analyses were conducted in IBM SPSS Statistics Version 22.0 (IBM Corp., Armonk, NY, United States) PROCESS MACRO (Hayes, 2017).

## Results

### Demographic Information and Bivariate Correlation

Participants were 924 college students with an average age of 20.2. Among them, 111 (12.2 %) were female male students, 197 (21.6%) were only-child, 394 (43.6%) were year-three students, 703 (77.2%) majored in arts and humanity, 82 (9%) were probable depression, and 190 (20.8%) were probable anxiety, and 69 (7.5%) were internet addiction. The bivariate correlations among analyzed variables ranged from -0.487 to 0.582 in magnitude. Table 1 displays the demographic information and bivariate correlations.

**TABLE 1:** Mean, standard deviation (SD), and bivariate correlation between analyzed variables.

	Mean (SD) *N* (%)	PR	PC	NC	SE	SS	RR	PD	IA	Age	Sex	Grade	Major
PR	2.818 (0.729)	1											
PC	2.072 (0.586)	−0.006	1										
NC	1.234 (0.549)	0.184**	0.159**	1									
SE	3.732 (0.774)	0.15**	0.217**	0.083*	1								
SS	5.356 (1.109)	−0.045	0.582**	0.037	0.321**	1							
RR	3.206 (0.525)	−0.261**	0.329**	−0.307**	0.01	0.386**	1						
PD	−0.004 (1.625)	0.22**	−0.275**	0.221**	−0.04	−0.345**	−0.487**	1					
IA	42.047 (13.470)	0.248**	−0.145**	0.261**	0.159**	−0.154**	−0.363**	0.498**	1				
Age	20.2 (2.513)	0.012	−0.103**	0.037	−0.025	−0.066	−0.007	0.046	0.001	1			
Sex	111 (12.2%)	0.123**	0.127**	0.031	0.111**	0.083*	−0.021	−0.056	−0.026	−0.002	1		
Grade	394 (43.6%)	0.037	−0.035	0.07*	−0.043	−0.09**	−0.079*	−0.002	−0.001	0.579**	0.075*	1	
Major	703 (77.2%)	−0.03	−0.082*	−0.023	−0.068*	−0.056	0.005	0.028	−0.03	−0.02*	−0.318**	−0.055	1
OC	197 (21.6%)	0.045	−0.04	−0.016	0.019	−0.019	0.018	0.017	0.01	0.084*	0.042	0.042	0.023

PR, the perceived risk of COVID-19; PC, positive coping style; NC, negative coping style; SE, self-esteem; SS, the perceived social support from teachers and family members; RR, roommate relationship; PD, psychological distress; IA, internet addiction; sex, female proportion; grade, the proportion of year three; major, the proportion of arts and humanity major; OC, the proportion of only-child; analyzed sample size ranged from 876 to 924. * p < 0.05; ** p < 0.01.

## Mediation

**FIGURE 2:**
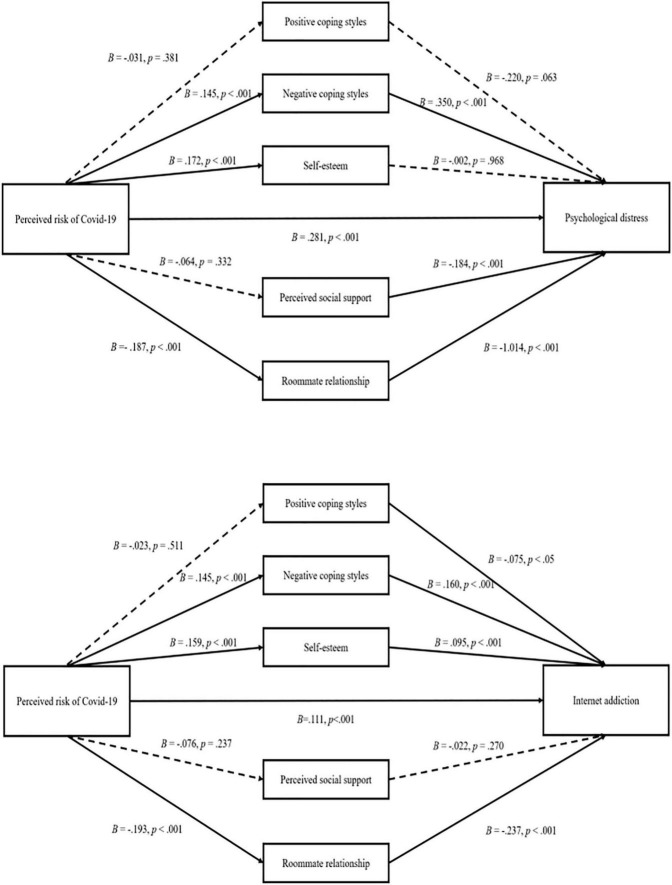
The parallel mediation between the perceived risk of COVID-19, psychological distress, and internet addition.

Table 2 and Figure 2 demonstrates the results of the parallel mediation. For personal resources, confirming H5, H7, and H10, the perceived risk of COVID-19 was positively associated with psychological distress *via* negative coping (β = 0.051, 95% *CI* 0.021 to 0.091) and internet addiction *via* negative coping and self-esteem (β = 0.023, 95% *CI* 0.012 to 0.039 for negative coping style, β = 0.015, 95% *CI* 0.006 to 0.027 for self-esteem). For social resources, confirming H4 and H9, the perceived risk of COVID-19 was positively associated with psychological distress and internet addiction *via* roommate relationships (β = 0.19, 95% *CI* 0.129 to 0.262 for psychological distress, β = 0.046, 95% *CI* 0.028 to 0.067 for internet addiction).

**TABLE 2:** The mediating effects of personal and social resource.

	Psychological distress *N* = 835		Internet addiction *N* = 853	
	β	95% *CI*	β	95% *CI*
Direct effect for perceived risk	**0.281**	**0.150 to 0.411**	**0.111**	**0.065 to 0.156**
**Indirect effect for personal resources**				
Positive coping	0.007	−0.009 to 0.029 (H1)	0.002	−0.004 to 0.009 (H6)
Negative coping	**0.051**	**0.021 to 0.091 (H5)**	**0.023**	**0.012 to 0.039 (H10)**
Self-esteem	−0.004	−0.022 to 0.022 (H2)	**0.015**	**0.006 to 0.027 (H7)**
**Indirect effect for social resources**				
Perceived social support	0.012	−0.012 to 0.041 (H3)	0.002	−0.002 to 0.008 (H8)
Roommate relationships	**0.190**	**0.129 to 0.262 (H4)**	**0.046**	**0.028 to 0.067**

*Age, sex, grade, only-child status, and major were used as controlled variables. Significant results are bolded.*

## Discussion

This is one of the first studies exploring the possible linking mechanism between perceived risk of COVID-19, psychological distress, and internet addiction using the stress-process model. Our findings informed clinicians and psychologists of two possible psychological mechanisms (negative coping style and roommate relationship) consistently existing in two outcomes.

Considering the personal resources, our results suggested that a higher level of perceived risk may suppress personal coping resources by boosting the negative coping styles, consistent with previous studies among the Chinese general population during COVID-19 (Wang et al., 2020). People having experienced post-traumatic experiences were more likely to adopt negative coping styles, which, in turn, is related to subsequent mental illness (Clarke and Goosen, 2009; Scheenen et al., 2017). On the other hand, people adopting positive coping styles tend to experience emotional wellbeing (Fredrickson and Joiner, 2002; Clarke and Goosen, 2009). Thus, college students with negative coping styles should be receiving more attention and short-term coping interventions should be urgently considered.

Given the social resources, the roommate relationship acts as a significant mediator. In China, most university students live with their roommates in dormitories and share the common experience of living on-campus. The zero-tolerance and lock-down policies in the local government force college students to stay in the dormitories all day long, which may amplify the roommate conflict, leading to psychological distress and internet addiction. In contrast, a good roommate relationship plays a vital role in mental health and adjustment to college life (Waldo and Fuhriman, 1981). Therefore, college students with poor roommate relationships should be given more attention and appropriate intervention should be delivered urgently.

Our findings did not support the mediating role of positive coping style and perceived social support, and partially support self-esteem. One possibility is that the positive coping styles are more likely associated with positive adjustment outcomes to stress. Thus, future studies should also incorporate outcomes (e.g., flourishing and post-traumatic growth) from positive psychology (Seligman and Csikszentmihalyi, 2014). Secondly, cultural differences regarding the meaning of self-esteem may explain the non-significant effects of psychological distress in the Chinese setting. In China, people may weigh more on collective self-esteem than an individual’s self-esteem due to the political system and Confucianism, thereby linking collective self-esteem with collective wellbeing (Zhang, 2005). Future studies should explore the interplay between collective and individual self-esteem and its relevance to psychological adjustment in the Chinese setting. Thirdly, college students have limited direct contact with their family members and teachers in the lock-down period, which might explain the null mediation effect of perceived social support from these people.

Some limitations should be addressed. First, the nature of the cross-sectional study cannot indicate the directionality, temporality, and causality of these variables. Second, we cannot rule out the roles of other personal (e.g., mastery) and social resources (e.g., family resilience). Third, most participants are female, art, and humanity majoring students, so our results cannot generalize to other settings. Fourth, the resulted sample from convenience sampling may not be generalizable to a larger sample of college students, especially those universities with more students majoring in science and engineering.

## Practical and Theoretical Implication

Our findings offer insights for future interventions and public health programs aiming at reducing psychological distress and internet addiction among college students who are quarantined on campus *via* altering negative coping styles and building up roommate relationships. Future longitudinal studies should also consider incorporating other resources (e.g., mastery or family resilience) in this theoretical model and explore the temporal relationships between these resources, which may provide a more comprehensive picture of the stress process.

## Conclusion

In sum, the perceived risk of COVID-19 is positively associated with psychological distress and internet addiction in college students who are experiencing a lock-down on campus. The possible mechanisms are negative coping styles and roommate relationships.

## Data Availability Statement

The raw data supporting the conclusions of this article will be made available by the authors, without undue reservation.

## Ethics Statement

This ethics study was reviewed and approved by the Institutional Review Board of Xi’an University by expediting mode. The patients/participants provided their written informed consent to participate in this study.

## Author Contributions

JH: conceptualization, writing, revisions, and data analysis. BC: writing, data collection, revisions, and funding. Both authors contributed to the article and approved the submitted version.

## Conflict of Interest

The authors declare that the research was conducted in the absence of any commercial or financial relationships that could be construed as a potential conflict of interest.

## Publisher’s Note

All claims expressed in this article are solely those of the authors and do not necessarily represent those of their affiliated organizations, or those of the publisher, the editors and the reviewers. Any product that may be evaluated in this article, or claim that may be made by its manufacturer, is not guaranteed or endorsed by the publisher.
